# Differentially Expressed Gene Annotator (DEGAn): automated annotation and analysis of DEGs datasets with OS and PFS data

**DOI:** 10.1093/bioadv/vbag128

**Published:** 2026-06-20

**Authors:** Giuseppe Agapito, Mario Cannataro

**Affiliations:** Department of Law, Economics and Sociology, University Magna Græcia of Catanzaro, Catanzaro, 88100, Italy; Data Analytics Research Center, University Magna Græcia of Catanzaro, Catanzaro, 88100, Italy; Laboratorio di Storia Giuridica ed Economica Research Center, University Magna Græcia of Catanzaro, Catanzaro, 88100, Italy; Cultura Romana del Diritto e Sistemi Giuridici Contemporanei Research Center, University Magna Græcia of Catanzaro, Catanzaro, 88100, Italy; Data Analytics Research Center, University Magna Græcia of Catanzaro, Catanzaro, 88100, Italy; Department of Medical and Surgical Sciences, University Magna Græcia of Catanzaro, Catanzaro, 88100, Italy

## Abstract

**Motivation:**

High-throughput transcriptomic platforms routinely generate large differentially expressed gene (DEG) datasets, but linking these matrices to clinical outcomes still often requires manual data integration and repeated gene-by-gene survival analyses. This limits scalability, reproducibility, and practical use in translational research.

**Results:**

We present DEGAn (Differentially Expressed Gene Annotator), a Java-based application for automated survival analysis of DEG matrices annotated with clinical data. DEGAn integrates gene expression with overall survival (OS) and progression-free survival (PFS), performs Kaplan–Meier and log-rank analyses across all genes in a single run, and returns ranked results with false discovery rate correction. The updated version extends DEGAn with configurable stratification strategies, alternative missing-value handling, multi-group survival analysis, and enhanced reporting to support sensitivity assessment and reproducible exploratory screening. DEGAn provides a graphical user interface and is distributed as a standalone executable for local, privacy-preserving analysis.

**Availability and implementation:**

DEGAn is implemented in Java and released under the LGPL v3.0+ license. Source code and binaries are available at https://gitlab.com/giuseppeagapito/degan.git.

## Introduction

RNA-seq, next-generation sequencing, and microarray platforms generate large-scale gene expression data that are routinely summarized as differentially expressed gene (DEG) matrices (Heller 2002, Marguerat and Bähler 2010, Wang and Karamyshev 2020). Although these data are widely used for biomarker discovery, their clinical interpretation remains limited when survival endpoints and other annotations must be integrated manually and analyzed one gene at a time (Brazma and Vilo 2000, Swanton et al. 2010). Such workflows are time-consuming, difficult to reproduce, and poorly suited to transcriptome-wide screening.

Kaplan–Meier survival analysis and log-rank testing are widely available in commercial, open-source, and web-based tools. However, most existing solutions are oriented toward single-gene or small-scale analyses, require substantial preprocessing, or depend on predefined public datasets (Chandrashekar et al. 2017, 2022, Posta and Győrffy 2025). As a consequence, systematic survival screening across user-defined DEG matrices remains cumbersome.

To address this limitation, we developed DEGAn (Differentially Expressed Gene Annotator), a standalone Java application for automated annotation and survival analysis of DEG datasets with OS and PFS information. DEGAn performs matrix-level survival analysis in a single execution, ranks genes according to statistical significance with false discovery rate adjustment, and supports local execution to reduce privacy risks associated with sensitive genomic and clinical data (Agapito and Cannataro 2023, Agapito et al. 2023).

## Background

Kaplan–Meier survival analysis is widely used to relate gene expression to outcomes such as overall survival (OS) and progression-free survival (PFS). Although commercial software, open-source statistical environments, and web-based portals support survival analysis, most existing solutions are geared toward single-gene or small-scale studies, often requiring manual preprocessing, repeated analyses, or programming expertise. These limitations reduce scalability, automation, and reproducibility when high-dimensional DEG matrices must be screened systematically.

To address this gap, we developed *DEGAn*, a standalone framework for automated survival analysis of annotated DEG matrices. DEGAn performs matrix-level analysis in a single run, integrating data harmonization, missing-value handling, expression stratification, and false discovery rate correction to produce ranked survival-associated genes. A broader comparison with existing tools and their limitations is provided in the Supplementary Material.

## Implementation

DEGAn is a platform-independent Java application that performs automated survival analysis of annotated DEG matrices. It integrates gene-expression and clinical data, performs one-click analysis of overall survival (OS) and progression-free survival (PFS), and provides interactive visualization of Kaplan–Meier curves for prioritized genes.

For each gene, DEGAn estimates survival curves and evaluates differences among expression-defined groups using Kaplan–Meier analysis and log-rank testing. DEGAn supports multiple stratification strategies, including median split, tertile-based grouping, quartile-based grouping, and manually defined thresholds. Different stratification strategies may capture distinct biological patterns of gene-expression effects on prognosis. Median dichotomization is widely used for its simplicity and interpretability, but it may miss non-linear effects or patterns visible only at expression extremes. Tertile- and quartile-based stratification can reveal more graded survival trends and better reflect patient heterogeneity, whereas manual thresholds may be preferable when prior biological or clinical knowledge suggests specific cutoffs. For missing data, mean imputation is a simple and computationally efficient default, but it has limitations: it reduces variance, may weaken biologically meaningful differences among samples, and can affect assignment to expression-based groups. In survival analysis, this may influence Kaplan–Meier separation and log-rank significance. For this reason, DEGAn also supports KNN-based imputation and complete-case analysis. Median-based grouping and simple imputation remain the defaults because they provide an accessible, fast, and easily interpretable baseline workflow, while more advanced methods are available when justified by data structure and missingness patterns.

Algorithm 1 summarizes the same configurable workflow described in the Supplementary Material, here reported in compact form.

Algorithm 1 DEGAn workflow for configurable transcriptome-wide survival analysis

Require: DEG matrix $X\in R^{G\times S}$ (genes × samples); clinical table *100* with survival time *T* and event indicator δ; stratification mode *M*; imputation mode *I*

Ensure: Ranked list of genes with log-rank statistics, adjusted *q*-values, missingness rates, and optional Kaplan–Meier curves

1: Load DEG matrix *X* and clinical table *C*

2: Harmonize sample identifiers and reorder columns of *X* to match *C*

3: Validate survival fields: ensure T≥0 and δ∈{0,1}

4: Initialize result container R

5: **for**  g←1 to *G* **do** 

6:   Compute missing-data rate $r_{g}$

7:   **if**  I= mean or median imputation **then** 

8:    Estimate the selected summary statistic over observed values of gene *g*

9:   Impute missing values of gene *g*

10:  **else if**  I= KNN imputation **then** 

11:   Impute missing values of gene *g* using KNN

12:  **else if**  I= complete-case analysis **then** 

13:   Remove samples with missing values for gene *g*

14:  **end if** 

15: **end for** 

16: P← number of available CPU cores

17: Partition gene indices {1,…,G} into disjoint blocks

18: **for all** gene blocks $B_{i}$  **in parallel do** 

19:  **for all**  $g\in B_{i}$  **do** 

20:   Define expression groups for gene *g* according to stratification mode *M*

21:   Construct survival bundles linking expression values with $\left(T_{s},\delta_{s}\right)$

22:   Estimate Kaplan–Meier curves for all detected groups

23:   Compute global log-rank test and raw *P*value $p_{g}$

24:   **if** number of groups >2  **then** 

25:    Compute pairwise post-hoc log-rank comparisons

26:   **end if** 

27:   Store gene identifier, $r_{g}$, $p_{g}$, group statistics, and survival summaries in R

28:   **end for** 

29: **end for** 

30: Apply Benjamini–Hochberg correction to obtain adjusted *q*-values

31: Rank genes by increasing *q*-values

32: Provide ranked results, missingness summaries, and Kaplan–Meier curves for visualization

To improve scalability, DEGAn processes genes independently in parallel on multi-core architectures. Results are ranked after false discovery rate correction, and significant genes can be explored interactively through survival-curve visualization. Additional implementation details are reported in the Supplementary Material.

## Performance evaluation

We evaluated DEGAn on synthetic DEG datasets with increasing dimensionality to characterize scalability and computational efficiency. Two matrix sizes were considered, corresponding to 20 000 and 100 000 genes, while the number of samples was progressively increased from 100 to 1000. Across all configurations, execution time increased smoothly with cohort size and remained only moderately affected by the five-fold increase in the number of genes. Energy consumption followed a similar trend, increasing approximately linearly with the number of samples. Memory usage remained bounded across all tested settings, indicating that DEGAn avoids large intermediate in-memory structures and is suitable for execution on standard workstations.

Overall, these results show that DEGAn scales efficiently with the width of the dataset while remaining robust to increases in feature dimensionality, supporting its use for transcriptome-wide exploratory survival analysis.

Extended validation results, detailed methods, algorithmic workflow, complexity analysis, and additional figures and tables are reported in the Supplementary Material.

## Algorithm complexity analysis

Let *G* denote the number of genes, *S* the number of samples, and *C* the number of available CPU cores. The preprocessing phase depends on the selected missing-data handling strategy. Simple mean/median imputation requires a linear scan of each gene and therefore costs O(GS) overall, whereas complete-case analysis also requires O(GS) time to identify and remove missing entries. KNN-based imputation is more computationally demanding; in the worst case, its cost depends on the number of incomplete samples and nearest-neighbor searches, leading to a higher preprocessing overhead than summary-statistic imputation.

For each gene, stratification requires at most a linear scan over *S* samples once the required cutoff values have been determined. Median-based grouping has linear cost, while tertile- and quartile-based grouping require quantile computation, which can be performed in O(S log S) time if based on sorting. Manual-threshold grouping remains linear in *S*. Survival analysis then requires construction of the expression-defined groups, estimation of Kaplan–Meier curves, and computation of the log-rank statistic. This step has per-gene complexity O(S log S) because event times must be ordered.

Accordingly, under the default workflow based on summary-statistic imputation and median stratification, the overall complexity remains O(GS log S). When quantile-based stratification is selected, the asymptotic cost is unchanged, since sorting dominates. When KNN imputation is used, total runtime increases by an additional preprocessing term reflecting nearest-neighbor computation, but the downstream survival-analysis stage remains unchanged.

If more than two expression-defined groups are generated, DEGAn first computes a global log-rank test and then performs pairwise post-hoc comparisons. For *K* groups, this introduces up to $O(K^{2})$ pairwise tests per gene; however, since *K* is small in practice (e.g., K=3 for tertiles and K=4 for quartiles), this does not change the dominant asymptotic term.

Since genes are independent, DEGAn parallelizes computation across $C^{\prime }\approx C-1$ workers, leading to an expected wall-clock time of $O\left(\frac{G}{C^{\prime }}S\;\text{log}\;S\right)$ under the default workflow, with modest synchronization overhead. Memory usage is dominated by storage of the input expression matrix and therefore scales as O(GS). Additional per-gene statistics, missingness information, and survival summaries are maintained using compact shared data structures, yielding stable memory consumption across increasing feature dimensionalities.

## Validation and discussion

DEGAn was validated on the public GEO dataset GSE30219, which contains gene expression profiles and overall survival information for lung cancer patients (Rousseaux et al. 2013). DEGAn directly processed the full dataset and produced a ranked list of genes associated with survival. Table 1 lists the first three top-ranked probes identified by DEGAn in the GSE30219 validation dataset. For validation, representative top-ranked probes were exported into an SPSS-compatible format and reanalyzed using Kaplan–Meier estimation and log-rank testing. The resulting survival curves and test statistics were consistent with those obtained using DEGAn, confirming the correctness of the implementation.

**Table 1:** Top probes identified by DEGAn in the GSE30219 validation dataset.

Probe ID	Gene	*p*-value
15,53,015_a_at	RECQL4	$1.0037\times 10^{-3}$
2,28,775_at	EMC3	$1.1620\times 10^{-3}$
15,52,680_a_at	KNL1	$1.4874\times 10^{-3}$

The current version improves methodological flexibility while preserving automation. Configurable stratification and imputation options enable users to assess the impact of analytical choices on survival results, while multi-group analysis broadens the range of supported study designs. At the same time, local execution and one-click matrix-level processing remain key practical advantages over manual gene-by-gene workflows and web-based systems restricted to predefined cohorts.

In the analyzed lung cancer dataset, DEGAn prioritized probes associated with genes such as *RECQL4*, *EMC3*, and *KNL1*, supporting its ability to highlight biologically and clinically meaningful candidates for downstream investigation (Wang et al. 2024, Tang et al. 2024, Li et al. 2026).

**Figure 1:**
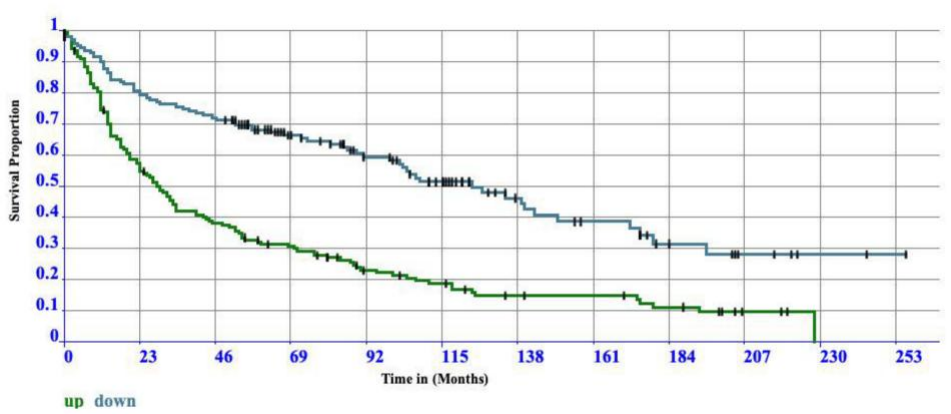
Overall survival curve generated by DEGAn for the probe set *15,52,680_a_at*.

**Figure 2:**
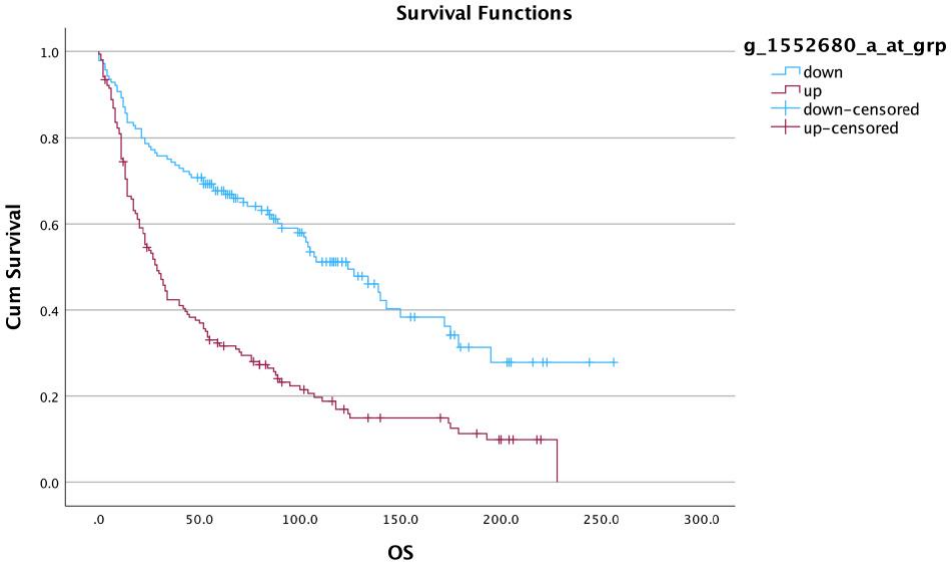
Corresponding overall survival curve generated by SPSS for the probe set *15,52,680_a_at*.

The close agreement between Figures 1 and 2 further confirms the consistency of DEGAn with SPSS statistical platform, while highlighting the practical advantage of transcriptome-wide automated analysis in a single executable workflow. Extended analyses, validation results, and additional figures and tables are provided in the Supplementary Material.

## Conclusions

DEGAn provides an automated framework for integrating DEG matrices with clinical survival data and performing transcriptome-wide OS and PFS analysis in a single workflow. By replacing repetitive gene-by-gene procedures with matrix-level processing, DEGAn improves scalability, reproducibility, and usability in translational research settings. The updated implementation further strengthens the software by introducing configurable stratification, alternative imputation strategies, multi-group survival analysis, and richer reporting for sensitivity assessment. DEGAn therefore represents a practical and extensible tool for survival-oriented analysis of transcriptomic data.

## Supplementary Material

vbag128_Supplementary_Data

## Data Availability

The data underlying this article are available in the Gene Expression Omnibus database at https://www.ncbi.nlm.nih.gov/geo/query/acc.cgi? acc=GSE30219 under accession number GSE30219.
