# Supplementary material for: Differentially Expressed Gene Annotator (DEGAn): automated annotation and analysis of DEGs datasets with OS and PFS data
Source: Bioinform Adv. 2026 Jun 20;6(1):vbag128. doi: 10.1093/bioadv/vbag128 (PMC13282708; doi:10.1093/bioadv/vbag128)
Supplement: vbag128_Supplementary_Data [file vbag128_supplementary_data.zip › DEGAnSupplementaries.pdf]

Bioinformatics, 2026, pp. 1–9
DOI added during production
Published: Date added during production
Supplementary Material

Supplementary Material for: Differentially Expressed Gene Annotator (DEGAn): Automated Annotation and Analysis of DEGs Datasets with OS and PFS Data

Giuseppe Agapito[1,3,4,5*] and Mario Cannataro[2,3]

[1]Department of Law, Economics and Social Sciences, University Magna Græcia of Catanzaro, Viale Europa, 88100, Catanzaro, Italy
[2]Department of Medical and Surgical Sciences, University Magna Græcia of Catanzaro, Viale Europa, 88100, Catanzaro, Italy
[3]Data Analytics Research Center, University Magna Græcia of Catanzaro, Viale Europa, 88100, Catanzaro, Italy
[4]Laboratorio di Storia Giuridica ed Economica Research Center, University Magna Græcia of Catanzaro, Viale Europa, 88100, Catanzaro, Italy
[5]Cultura Romana del Diritto e Sistemi Giuridici Contemporanei Research Center, University Magna Græcia of Catanzaro, Viale Europa, 88100, Catanzaro, Italy

*Corresponding author. agapito@unicz.it

Abstract

Motivation: High-throughput technologies such as RNA-seq, next-generation sequencing, and microarray platforms routinely generate large-scale differential expression results. However, the association of differentially expressed genes (DEGs) with clinical outcomes still relies on manual integration of heterogeneous clinical tables and repeated per-gene survival analyses. These workflows are time-consuming, poorly scalable, and difficult to reproduce, limiting their applicability in translational and clinical research.
Results: We present DEGAn (Differentially Expressed Gene Annotator), a Java-based application that automates survival analysis on DEG matrices. DEGAn integrates gene expression data with clinical endpoints, including overall survival and progression-free survival, and performs Kaplan–Meier estimation and log-rank testing across the entire annotated dataset in a single run. The tool produces a ranked list of genes based on statistical significance, with false discovery rate adjustment, enabling rapid and systematic prioritization of clinically relevant genes without iterative, manual per-gene testing. DEGAn provides an interactive graphical user interface for data integration and visualization and is distributed as a standalone executable `.jar`, facilitating local execution and integration into analysis pipelines.
Availability and implementation: DEGAn is implemented in Java and released under the LGPL v3.0+ license. Source code and binaries are available at `https://gitlab.com/giuseppeagapito/degan.git`. **Contact: agapito@unicz.it Supplementary information: Supplementary data are available at Bioinformatics online.**

Extended background and related tools

This section provides an extended description of representative commercial, open-source, and web-based tools supporting Kaplan–Meier survival analysis. The material reported here complements the concise overview presented in Section Background of the main manuscript and is included to support transparency and reproducibility.

- GraphPad PRISM[1] is widely adopted in the biomedical and life sciences. A principal strength is its intuitive graphical interface, which enables even researchers lacking extensive statistical or programming expertise to perform statistical analysis. Common statistical analyses -t-tests, ANOVA, nonlinear regression, and survival analysis- are implemented through guided workflows that could minimize procedural errors. To limit inconsistencies and manual transcription errors, GraphPad automatically updates figures as data is adjusted. Finally, GraphPad is supported by extensive documentation and tutorials, enhancing its accessibility. Despite these advantages, GraphPad has some limitations that hinder its use in advanced or large-scale analytical contexts. Prism is optimized for small to moderate-sized datasets typical of laboratory experiments. Performance and usability decrease with large datasets, complex relational data structures, or multi-source data integration. Additionally, licensing costs may pose a barrier for some institutions or individual researchers, especially in comparison to freely available statistical software. GraphPad has several limitations for computing survival analyses from datasets of differentially expressed genes (DEGs). First, each DEG row—corresponding to a single gene—must be analyzed individually, making it impossible to identify statistically relevant genes a priori without performing OS analysis for all genes in the dataset. This row-by-row approach substantially limits scalability and increases analytical overhead. Moreover, for each gene, survival time and event status must be manually reformatted

[1] https://www.graphpad.com/features

© The Author(s) 2026. Copyright and licence statements to be updated by the publisher during production.

into column-based structures and pasted into the first two Prism columns (X for time and Y for event), introducing a labor-intensive step that is prone to transcription errors and hinders reproducibility. In conclusion, although suitable for interactive, exploratory analysis, it lacks native methods for effectively analyzing DEGs in OS studies and scripting capabilities, making it less appropriate for high-throughput analyses, batch processing, or integration into automated workflows. As a result, achieving version control and programmatic reproducibility is more challenging.

- SPSS (IBM SPSS Statistics)[2] is widely used in biomedical, clinical, and epidemiological research. SPSS provides native support for a broad range of statistical procedures, including descriptive statistics, hypothesis testing, regression models, and survival analysis. Kaplan–Meier estimators, log-rank tests, and Cox proportional hazards models are implemented through structured dialogs that guide users through parameter specification, reducing the risk of procedural errors. Extensive documentation and institutional adoption further contribute to its widespread use in clinical research environments. Despite these advantages, SPSS exhibits several limitations that restrict its effectiveness in advanced or large-scale analytical contexts. Although capable of handling moderately large datasets, performance and usability decline when dealing with high-dimensional data, complex relational structures, or multi-source data integration. Moreover, as a proprietary platform, licensing costs may limit accessibility for some institutions or individual researchers, particularly when compared to open-source statistical alternatives. SPSS presents specific limitations in computing survival analyses from datasets of differentially expressed genes (DEGs). Each DEG—corresponding to a single gene—must be treated as an independent variable, requiring iterative survival modeling across the entire gene set to identify statistically relevant associations with overall survival (OS). As a result, it is not possible to preselect relevant genes a priori without performing OS analysis for all DEGs, substantially limiting scalability. Furthermore, survival time and event status must be correctly structured and repeatedly associated with each gene expression vector, necessitating extensive data reshaping and complex syntax-based loops. This workflow is labor-intensive, computationally inefficient, and poorly suited to high-throughput transcriptomic analyses. In conclusion, while SPSS is well-suited for structured clinical survival analyses and hypothesis-driven modeling, it lacks native support for omics-oriented workflows and automated DEG-to-survival pipelines. Consequently, its applicability to large-scale OS screening studies is limited, and integration into fully automated, reproducible bioinformatics workflows remains challenging.
- MedCalc[3]] is a statistical software package primarily designed for biomedical and clinical research, with a strong emphasis on medical statistics and diagnostic test evaluation. A principal strength of MedCalc is its user-friendly graphical interface, which enables clinicians and biomedical researchers with limited programming expertise to perform standard statistical analyses efficiently. The software provides native support for commonly used statistical methods, including descriptive statistics, hypothesis testing, regression models, receiver operating characteristic (ROC) curve analysis, and survival analysis. Kaplan–Meier survival curves and log-rank tests are implemented through guided procedures that simplify parameter selection and reduce procedural errors. MedCalc is supported by extensive documentation and tutorials tailored to medical applications, which enhance its accessibility in clinical research settings. Despite these advantages, MedCalc presents several limitations that constrain its applicability in advanced or large-scale analytical contexts. The software is optimized for small to moderate-sized clinical datasets and performs less effectively on high-dimensional or complex data. Additionally, as proprietary software, MedCalc requires a commercial license, which may represent a barrier for some users when compared to freely available statistical tools. MedCalc has specific limitations in computing survival analyses from datasets of differentially expressed genes (DEGs). Each DEG—corresponding to a single gene—must be analyzed individually, requiring repetitive survival analyses across the entire gene set to identify statistically significant associations with overall survival (OS). This gene-by-gene approach prevents the identification of relevant genes a priori and substantially limits scalability. Moreover, survival time and event status must be manually aligned with each gene expression vector, requiring repeated data restructuring and manual intervention. This workflow is labor-intensive, prone to transcription errors, and unsuitable for high-throughput transcriptomic analyses. In conclusion, although MedCalc is well-suited for clinical and diagnostic survival analyses in small, well-defined datasets, it lacks native support for omics-oriented workflows, scripting capabilities, and automated DEG-to-survival pipelines. Consequently, its use in large-scale OS screening studies and integration into reproducible, automated bioinformatics workflows is limited.

Follows a list of open-source tools that include GUI, scripting, and web-based interaction modes, along with their principal analysis features.

- EZR (Easy R)[4]] is a statistical software interface based on the R environment, specifically developed to support biostatistical analyses in medical research. A principal strength of EZR is its capacity to combine the analytical power and extensibility of R with a simplified graphical user interface, making advanced statistical methods accessible to users with limited programming expertise. EZR provides native support for a wide range of statistical procedures commonly used in clinical research, including descriptive statistics, hypothesis testing, regression models, and survival analysis. Kaplan–Meier estimation, log-rank tests, and Cox proportional hazards models are implemented through guided dialog boxes, reducing procedural complexity while retaining methodological rigor. EZR's graphical interface makes standard analyses easier, but its usability decreases with very large or complex datasets. The GUI can hide underlying R code, limiting advanced customization unless users work directly with R scripts. Additionally, although EZR simplifies many tasks, complex analyses often require familiarity with R, which may limit accessibility for users without programming experience. EZR has limitations when computing survival analyses from datasets of

[2] https://www.ibm.com/it-it/products/spss-statistics
[3] [https://www.medcalc.org/en/
[4] [https://www.jichi.ac.jp/usr/hema/EZR/statmedEN.html

differentially expressed genes (DEGs). Each DEG—corresponding to a single gene—must be analyzed individually, requiring iterative survival modeling across the entire gene set to identify statistically relevant associations with overall survival (OS). As a consequence, it is not possible to identify relevant genes a priori without performing OS analyses for all DEGs, limiting scalability in transcriptomic studies. Moreover, survival time and event status must be repeatedly and correctly associated with each gene expression vector, necessitating extensive data restructuring and, in many cases, manual intervention through the GUI. While scripting in R can mitigate some of these limitations, such functionality lies outside EZR's primary GUI-driven workflow. In conclusion, although EZR represents a valuable compromise between usability and statistical rigor for clinical survival analyses, it lacks native, streamlined support for high-throughput DEG-based OS screening and fully automated omics pipelines. Consequently, its integration into large-scale, reproducible bioinformatics workflows remains limited without substantial customization through direct R programming.

- JASP[5] is an open-source statistical software designed to provide statistical analysis in the social and biomedical sciences. A principal strength of JASP is its intuitive graphical interface, which enables researchers without programming expertise to perform standard statistical analyses efficiently. JASP comes with several statistical procedures, including descriptive statistics, hypothesis testing, regression models, and Bayesian inference. Survival analysis functionality, including Kaplan–Meier curves and log-rank tests, is available through dedicated modules, implemented via guided workflows that reduce procedural complexity. As an open-source tool, JASP benefits from community-driven development. Despite these advantages, JASP exhibits several limitations that constrain its applicability in advanced or large-scale analytical contexts. The software is primarily optimized for interactive, GUI-driven analyses on small to moderate-sized datasets. Performance and flexibility decrease when dealing with high-dimensional data, complex data transformations, or multi-source data integration. Moreover, JASP offers limited scripting and automation capabilities, which limit its suitability for batch processing and large-scale analytical pipelines, despite its reliance on established statistical engines. JASP presents specific limitations in computing survival analyses from datasets of differentially expressed genes (DEGs). Each DEG—corresponding to a single gene—must be analyzed individually, requiring repetitive survival analyses across the entire gene set to identify statistically relevant associations with overall survival (OS). This gene-by-gene approach prevents the identification of relevant genes a priori and substantially limits scalability. Furthermore, survival time and event status must be repeatedly and manually associated with each gene expression vector via the graphical interface, resulting in labor-intensive workflows prone to transcription errors and unsuitable for high-throughput transcriptomic analyses. In conclusion, although JASP is well-suited for experimental survival analyses and educational or hypothesis-driven studies, it lacks native support for omics-oriented workflows, automated DEG-to-survival pipelines, and programmatic control. Consequently, its integration into large-scale, reproducible bioinformatics workflows and high-throughput OS screening studies remains limited.
- jamovi[6] is an open-source, GUI-based statistical software built on top of the R ecosystem, designed to provide an intuitive and transparent environment for statistical analysis in the social and biomedical sciences. A principal strength of jamovi lies in its user-friendly interface, combined with direct integration of validated R packages, which allow users to perform advanced statistical analyses without writing code. The software supports a wide range of standard statistical procedures, including descriptive statistics, hypothesis testing, regression models, and survival analysis. Through dedicated modules such as ClinicoPath/jsurvival, jamovi provides access to Kaplan–Meier survival curves, log-rank tests, and Cox proportional hazards models via guided workflows that reduce procedural complexity and improve accessibility for non-programmers. Automatic updating of results and figures as data are modified further enhances analytical consistency and reduces manual transcription errors. Despite these advantages, jamovi has limitations that limit its applicability in advanced or large-scale analytical contexts. The software is primarily optimized for interactive, exploratory analyses on small to moderate-sized datasets. Performance and usability may degrade when handling high-dimensional data or complex data transformations typical of omics studies. Although jamovi is extensible through R-based modules, advanced customization and troubleshooting often require direct interaction with underlying R code, partially negating the benefits of the graphical interface. Moreover, the modular ecosystem depends on external package maintenance, which may introduce variability in long-term stability. jamovi, including the ClinicoPath/jsurvival module, has specific limitations in computing survival analyses from datasets of differentially expressed genes (DEGs). Each DEG—corresponding to a single gene—must be analyzed individually, requiring repetitive survival analyses across the entire gene set to identify statistically relevant associations with overall survival (OS). This gene-by-gene approach prevents the identification of relevant genes a priori and substantially limits scalability. Furthermore, survival time and event status must be repeatedly associated with each gene expression vector through the GUI, necessitating extensive data restructuring and manual intervention. While underlying R scripting could, in theory, enable automation, this functionality is not natively supported in jamovi's standard workflow. In conclusion, although jamovi and the ClinicoPath/jsurvival module represent an effective compromise between usability and statistical rigor for clinical survival analyses, they lack native support for high-throughput DEG-based OS screening and fully automated omics pipelines. Consequently, their integration into large-scale, reproducible bioinformatics workflows remains limited without substantial customization beyond the graphical interface.
- R[7] is an open-source programming language and statistical computing environment widely adopted in biomedical research, bioinformatics, and data science. A principal strength of R lies in its flexibility, extensibility, and comprehensive ecosystem of packages specifically developed for statistical modeling and high-throughput data analysis. R provides robust

[5] https://jasp-stats.org

[6] https://www.jamovi.org

[7] https://www.r-project.org

native support for survival analysis through well-established libraries, enabling Kaplan–Meier estimation, log-rank testing, and Cox proportional hazards modeling with fine-grained control over model specification. In contrast to GUI-based tools, R allows direct manipulation of complex data structures and supports fully scriptable workflows, facilitating automation, version control, and exact reproducibility. Extensive community-driven documentation and peer-reviewed package development further enhance its reliability and methodological transparency. Despite these advantages, R has a steeper learning curve than graphical statistical software. Effective use requires proficiency in programming concepts, data structures, and package management, which may represent a barrier for researchers without computational training. Additionally, the flexibility of R places greater responsibility on the user to ensure correct implementation of statistical methods, increasing the risk of user-induced errors in the absence of rigorous validation or standardized workflows. Performance may also depend on user expertise in optimizing code and managing memory when handling very large datasets. R exhibits notable strengths—but also practical challenges—in the computation of survival analyses from datasets of differentially expressed genes (DEGs). Unlike GUI-driven tools, R enables vectorized and programmatic survival modeling across entire DEG matrices, allowing statistically relevant genes to be identified a priori through automated screening procedures. However, implementing such pipelines requires advanced scripting, careful data preprocessing, and integration of multiple specialized packages. The absence of a unified, standardized DEG-to-survival framework can lead to heterogeneous analytical approaches across studies, potentially affecting comparability and reproducibility if workflows are not rigorously documented. In conclusion, R is highly suitable for large-scale OS analyses, high-throughput DEG screening, and integration into automated bioinformatics pipelines. Nevertheless, its effective use in this context depends strongly on programming expertise and methodological rigor, which may limit accessibility for users seeking interactive, GUI-based exploratory analysis without substantial computational investment.

- Kaplan–Meier Plotter[8] is a web-based analytical platform designed for evaluating the association between gene expression and survival outcomes in cancer studies. A principal strength of Kaplan–Meier Plotter lies in its immediate applicability to transcriptomic survival analysis, as it integrates large, pre-curated public datasets with built-in survival endpoints. The platform enables users to generate Kaplan–Meier survival curves, compute log-rank statistics, and explore hazard ratios through a streamlined web interface that requires no programming expertise. By automating data preprocessing, patient stratification, and survival computation, Kaplan–Meier Plotter substantially lowers the technical barrier for exploratory OS analysis and allows rapid hypothesis generation in oncogenomic research. Kaplan–Meier Plotter Despite these advantages, Kaplan–Meier Plotter presents several limitations that restrict its applicability in advanced or customizable analytical contexts. The platform operates as a closed, web-based environment with limited transparency regarding underlying data preprocessing steps and analytical parameterization. Users are constrained to predefined datasets, gene annotations, and stratification strategies, reducing flexibility and limiting applicability beyond the supported cancer types and cohorts. Furthermore, integration with external datasets or user-generated DEG matrices is not natively supported, and analytical workflows cannot be exported or version-controlled programmatically. Kaplan–Meier Plotter exhibits specific limitations in the context of systematic survival analyses from datasets of differentially expressed genes (DEGs). Although the platform allows rapid evaluation of individual genes, each DEG—corresponding to a single gene—must be queried separately, preventing large-scale, automated OS screening across entire DEG lists. As a result, statistically relevant genes cannot be identified a priori without manually repeating analyses for all genes of interest. This one-gene-at-a-time approach substantially limits scalability and introduces potential selection bias in exploratory analyses. Additionally, the lack of scripting capabilities and batch-processing functionality hinders reproducibility and makes comprehensive DEG-level survival assessment impractical. In conclusion, while Kaplan–Meier Plotter is highly effective for rapid, exploratory survival analysis of individual genes using curated public datasets, it lacks the flexibility, transparency, and automation required for high-throughput DEG-based OS studies. Consequently, its integration into reproducible, large-scale bioinformatics workflows and systematic survival screening pipelines remains limited.
- GEPIA/GEPIA2 (Gene Expression Profiling Interactive Analysis)[9] is a web-based platform designed to facilitate analysis of gene expression and survival data derived from large-scale cancer genomics resources, primarily TCGA and GTEx. A principal strength of GEPIA/GEPIA2 lies in its seamless integration of uniformly processed RNA-seq data from these consortia, enabling rapid comparison between tumor and normal tissues without requiring local data preprocessing. The platform provides user-friendly modules for differential expression, correlation, and survival analyses, including Kaplan–Meier curves with log-rank testing. Through its intuitive web interface, GEPIA/GEPIA2 allows researchers without programming expertise to investigate associations between gene expression values and overall survival (OS), making it particularly useful for hypothesis generation and exploratory oncogenomic studies. Despite these advantages, GEPIA/GEPIA2 presents several limitations that restrict its applicability in advanced or customizable analytical contexts. As a web-based, closed platform, it offers limited transparency and control over underlying normalization procedures, cutoff definitions, and model parameterization. Users are constrained to predefined datasets, cancer types, and analytical options, with no native support for uploading external datasets or integrating user-generated DEG results. Moreover, analytical workflows cannot be scripted, exported, or version-controlled, limiting reproducibility and methodological flexibility. GEPIA/GEPIA2 exhibits specific limitations in the context of survival analyses from datasets of differentially expressed genes (DEGs). Although survival analysis is natively supported, each DEG—corresponding to a single gene—must be evaluated individually, preventing automated, large-scale OS screening across entire DEG lists. Consequently, it is not possible to identify statistically relevant

[8] https://kmplot.com/analysis/index.php?p=home

[9] http://gepia.cancer-pku.cn

genes a priori without manually repeating survival analyses for all genes of interest. This one-gene-at-a-time approach substantially limits scalability and introduces potential bias in gene selection. Additionally, the inability to adjust survival models beyond basic stratification further restricts its suitability for systematic DEG-level survival assessment. In conclusion, while GEPIA/GEPIA2 is well-suited for rapid, exploratory survival analysis using TCGA/GTEx data, it lacks the flexibility, automation, and transparency required for high-throughput DEG-based OS studies. As a result, its integration into reproducible, large-scale bioinformatics workflows and systematic survival screening pipelines remains limited.

To address the limitations shared by existing KM analysis tools, we present *DEGAn*, a software framework specifically designed for survival analysis of differentially expressed gene (DEG) datasets. Unlike general-purpose statistical platforms and web-based portals, which require each DEG—corresponding to a single annotated row—to be analyzed independently, DEGAn natively operates on fully annotated DEG matrices. This design eliminates the need for manual, gene-by-gene survival testing and enables systematic, high-throughput OS screening across all genes within a single workflow.

DEGAn provides: *(i)* an intuitive graphical interface to integrate not annotated DEGs with clinical data through visual commands, with automated harmonization of identifiers, data cleaning, handling of missing values, and normalization of time units; *(ii)* simultaneous analysis of all annotated DEGs, producing a ranked list of genes based on statistical significance, which overcomes the fundamental scalability limitation of existing KM tools that require repeated per-gene analyses and allows researchers to immediately identify genes most likely involved in the disease phenotype under investigation, without manual, iterative survival testing; *(iii)* automatic inference and validation of event times and censoring indicators; *(iv)* automated stratification of samples into up- and down-regulated groups using a median-based gene-expression filter; and *(v)* transparent application of false discovery rate (FDR) multiple-testing correction, yielding statistically robust overall-survival (OS) estimates for each DEG.

Finally, *(vi)* DEGAn is distributed as a standalone `.jar` executable, enabling seamless integration into automated analysis pipelines and facilitating programmatic scripting for advanced users. This deployment model distinguishes DEGAn from most GUI- and web-based KM tools, which lack native support for pipeline integration and reproducible, script-driven execution. Combined with *(vii)* built-in multi-core parallelism for efficient distribution of independent gene–outcome evaluations, DEGAn bridges the gap between interactive KM visualization tools and fully automated, reproducible bioinformatics workflows. Table 1 provides a concise comparison of the investigated software tools.

Data organization and methods

Gene expression and clinical data formats

Clinical data are provided in tabular form, where columns represent samples (or tissues) and rows encode temporal and clinical variables (Table 2). Each cell at row i and column j stores the time-to-event value t_i associated with subject j.

Overall survival (OS) measures the time until an event of interest occurs, while progression-free survival (PFS) measures the time to disease progression or recurrence. Both are associated with binary status indicators. PFS is meaningful only for tumor-bearing samples and is therefore complemented by the *Metastatic* variable.

Differentially expressed gene (DEG) datasets are organized in tabular form, where rows correspond to genes (probes), columns to samples, and each cell (i, j) contains the expression value of gene i in sample j (Table 3). This format is directly produced by standard high-throughput transcriptomic platforms, and the resulting table includes all genes (probes) measured by the platform, without prior feature-level filtering.

Overall and progression-free survival computation

Overall survival (OS) and progression-free survival (PFS) are estimated using the Kaplan–Meier (KM) product-limit estimator (3). Let $t_1 < t_2 < \cdots < t_K$ denote the ordered distinct event times. At each time t_k, let n_k be the number of subjects at risk immediately prior to t_k and d_k the number of observed events. Censored observations reduce the risk set after the censoring time and do not contribute to the event count.

The Kaplan–Meier estimate of the survivor function at time t is defined as

$$\hat{S}(t) = \prod_{k:t_k \le t} \left(1 - \frac{d_k}{n_k}\right). \quad (1)$$

Differences between survival distributions of expression-defined groups are assessed using the log-rank test. Under the null hypothesis of identical survival curves, the test statistic follows a chi-square distribution with $(j - 1)$ degrees of freedom for j groups and is computed as

$$\chi^2 = \sum_{i=1}^{j} \frac{(O_i - E_i)^2}{E_i}, \quad (2)$$

where O_i and E_i denote the observed and expected number of events in the i-th group, respectively.

Implementation

DEGAn is implemented in Java and is platform-independent through the Java Virtual Machine. Its core functionalities include: *(i)* automatic annotation of DEG matrices with clinical metadata (e.g., temporal events) and computation of overall survival (OS) and progression-free survival (PFS); *(ii)* one-click, dataset-wide survival analysis; and *(iii)* interactive visualization of OS and PFS curves stratified by expression-defined DEG groups, enabling prioritization of genes associated with the phenotype under investigation.

Survival estimation is performed using the Kaplan–Meier estimator (3), and differences between expression-defined groups are evaluated using the chi-square (χ^2) statistic through the log-rank test. DEGAn automatically infers gene-expression groups from the input DEG matrix, enabling efficient computation of OS and PFS curves without manual intervention.

In the updated implementation, DEGAn supports multiple stratification strategies, including median split, tertile-based grouping, quartile-based grouping, and manually defined thresholds. Different stratification strategies may capture distinct biological patterns of gene-expression effects on prognosis. Median dichotomization remains a widely used default because of its simplicity, interpretability, balanced group sizes, and reduced sensitivity to extreme values. Related median- or quantile-based normalization and grouping

Tool	GUI	DEGOpt	SGene	MGene	Scal	PipeReady	StandAl	OpenSo	MultTest
GraphPad PRISM	✓	×	✓	×	×	×	✓	×	×
SPSS	✓	×	✓	×	×	±	✓	×	±
MedCalc	✓	×	✓	×	×	×	✓	×	×
EZR (Easy R)	✓	×	✓	×	×	±	✓	✓	±
JASP	✓	×	✓	×	×	×	✓	✓	×
jamovi + jsurvival	✓	×	✓	×	×	±	✓	✓	±
R	×	×	✓	✓	✓	✓	✓	✓	✓
Kaplan–Meier Plotter	✓	×	✓	×	×	×	×	×	×
GEPIA / GEPIA2	✓	×	✓	×	×	×	×	×	×
**DEGAn**	✓	✓	✓	✓	✓	✓	✓	✓	✓

Table 1 Feature comparison of Kaplan–Meier survival analysis tools with respect to interface, DEG-specific optimization, scalability, and automation. ✓ denotes native support; ± indicates partial or indirect support requiring manual intervention or external scripting; × denotes absence of the feature. DEG optimization refers to native, matrix-level survival analysis of differentially expressed genes with ranked statistical output and multiple-testing correction. Pipeline-ready indicates suitability for integration into automated, programmatic analysis workflows.Comparison of survival analysis tools with respect to usability and support for DEG-based analyses. **GUI**: graphical user interface; **DEGOpt**: native support for differentially expressed gene (DEG) matrices; **SGene**: single-gene survival analysis only; **MGene**: multi-gene or transcriptome-wide survival analysis; **Scal**: scalability to high-dimensional datasets; **PipeReady**: suitability for automated pipeline integration; **StandAl**: availability as a standalone, locally executable application; **OpenSo**: open-source software; **MultTest**: native support for multiple-testing correction.

Table 2 Example of clinical data organization. S denotes samples. OS and PFS represent overall and progression-free survival times, respectively. Status variables are binary, with 1 indicating event occurrence and 0 censoring. The *Metastatic* variable indicates the presence (1) or absence (0) of metastasis.

	s_1	s_2	s_3	$\cdots$	s_N
OS	**26.6**	**15.7**	**32.2**	$\cdots$	**2.3**
StatusOS	**1**	**1**	**0**	$\cdots$	**1**
PFS	**16.6**	**4.7**	**3.8**	$\cdots$	**27.3**
StatusPFS	**1**	**0**	**0**	$\cdots$	**1**
Metastatic	**1**	**0**	**0**	$\cdots$	**1**

Table 3 Example of a DEG matrix. G and S denote gene and sample identifiers, respectively. Each cell (i, j) contains the expression value of gene i in sample j.

	s_1	s_2	s_3	$\cdots$	s_N
G_1	**235.523**	**307.569**	**196.315**	$\cdots$	**120.529**
G_2	**95.7887**	**41.3345**	**69.3994**	$\cdots$	**25.9387**
$\vdots$	$\vdots$	$\vdots$	$\vdots$	$\ldots$	$\vdots$
G_M	**84.8689**	**25.9345**	**80.7475**	$\cdots$	**128.917**

principles are implicitly employed in established DEG frameworks such as DESeq2 (6), edgeR (1), and EBSeq (4), enabling fair and robust comparisons across samples. In the context of survival analysis, median-based stratification provides a simple, transparent, and reproducible baseline for defining expression groups required for Kaplan–Meier modeling and log-rank testing (7). At the same time, tertile- and quartile-based grouping can reveal more graded survival trends and may better reflect biological heterogeneity among patients, whereas manual thresholds may be appropriate when prior biological or clinical knowledge suggests specific cutoffs.

Missing data handling has also been extended. In addition to simple mean/median imputation, DEGAn now supports KNN-based imputation and complete-case analysis. Mean imputation remains a computationally efficient default baseline, but it may reduce variance, attenuate biologically meaningful differences among samples, and influence patient assignment to expression-defined groups. In survival analysis, these effects may alter Kaplan–Meier separation and log-rank significance. For this reason, alternative approaches are provided to support sensitivity analysis and to adapt the workflow to the structure and missingness pattern of the data. DEGAn also reports the per-gene missing-data rate to support transparent analytical decisions.

Group detection and survival computation are executed in parallel by exploiting multi-core CPU architectures. Since DEG rows are independent, the input matrix is partitioned row-wise and processed concurrently, reducing execution time and energy consumption. Algorithm 1 summarizes the main steps of the updated DEGAn workflow.

As a first step, DEGAn determines the number of available CPU cores by invoking an appropriate operating system call and assigns bundles of rows accordingly (see lines 13–14 of Algorithm 1) to ensure a balanced distribution of the workload across computational units. Then, the obtained number of CPU cores, denoted as $nWorkers$, is used to split the DEG table. Specifically, the DEG table is virtually partitioned by the *Master* node into $nWorkers$ horizontal strips, each containing approximately $\frac{np}{nWorkers-1}$ genes (rows) and all columns (i.e., subjects and associated clinical data). In particular, each partition i, for $i = 1, \ldots, (nWorkers - 1)$, is identified by pointers to its first and last rows, denoted as $start_i$ and end_i, respectively. This strategy enables an even division of the DEG table across available computational resources, facilitating effective workload balancing and optimal resource utilization.

Afterwards, the *Master* instantiates $nWorkers - 1$ instances of node *workers*. Using $nWorkers - 1$ prevents system overloading, limiting the number of possible race conditions. After the split phase, each *worker* can analyze independently and iteratively each row belonging to its bundle to verify whether or not there are differences in the survival experiences of the detected groups (see Algorithm 1, lines 15–24). Depending on the selected configuration, genes may be grouped by median split, tertiles, quartiles, or user-defined thresholds, and stored in an ad-hoc data structure called *Bundle*, i.e., group.

The partitioning strategy used to classify DEGs depends on the selected stratification mode. In the default binary setting, let deg_i denote the expression value of the i-th gene, and let m_g be the median expression value computed for gene g. Each sample is assigned to one

Algorithm 1 DEGAn end-to-end workflow for configurable large-scale survival analysis

Require: DEG matrix $X \in \mathbb{R}^{G \times S}$ (genes $\times$ samples); clinical table C with survival time T and event indicator δ; stratification mode M; imputation mode I
Ensure: Ranked list of genes with log-rank statistics, adjusted q-values, missingness rates, and optional Kaplan–Meier curves
1: Load DEG matrix X and clinical table C
2: Harmonize sample identifiers and reorder columns of X to match C
3: Validate survival fields: ensure $T \geq 0$ and $\delta \in \{0, 1\}$
4: Initialize a thread-safe result container $\mathcal{R}$
5: **for** $g \leftarrow 1$ to G **do**
6: Compute missing-data rate r_g
7: **if** $I =$ mean or median imputation **then**
8: Estimate the selected summary statistic over observed values of gene g
9: Impute missing values of gene g using the selected statistic
10: **else if** $I =$ KNN imputation **then**
11: Impute missing values of gene g using KNN
12: **else if** $I =$ complete-case analysis **then**
13: Remove samples with missing values for gene g
14: **end if**
15: **end for**
16: $P \leftarrow$ number of available CPU cores
17: Partition gene indices $\{1, \ldots, G\}$ into $P' = \max(1, P - 1)$ disjoint blocks
18: **for all** gene blocks $\mathcal{B}_i$ **in parallel do**
19: **for all** $g \in \mathcal{B}_i$ **do**
20: Define expression groups for gene g according to stratification mode M
21: Construct survival bundles linking expression values with (T_s, δ_s)
22: Estimate Kaplan–Meier curves for all detected groups
23: Compute global log-rank test and raw p-value p_g
24: **if** number of groups > 2 **then**
25: Compute pairwise post-hoc log-rank comparisons
26: **end if**
27: Store gene identifier, r_g, p_g, group statistics, and survival summaries in $\mathcal{R}$
28: **end for**
29: **end for**
30: Apply Benjamini–Hochberg correction to obtain adjusted q-values
31: Rank genes by increasing q-values
32: Provide ranked results, missingness summaries, and Kaplan–Meier curves for visualization

of two regulation groups according to the following rule:

$$s(deg_i) = \begin{cases} \text{downReg}, & \text{if } deg_i \leq m_g, \\ \text{upReg}, & \text{if } deg_i > m_g. \end{cases} \tag{3}$$

More generally, DEGAn extends this rule to tertile- and quartile-based stratification by replacing the single median cut-point with the corresponding empirical quantiles, while manual-threshold mode assigns samples according to user-defined expression cutoffs.

To empirically assess robustness with respect to cutoff selection, DEGAn supports cutoff-sensitivity analysis at the DEG-matrix level. Gene separation obtained through Equation 3 remained consistent across alternative cutoff definitions, indicating that the observed associations were not driven solely by the specific choice of the median threshold (Figure 1). Accordingly, DEGAn enables systematic cutoff-sensitivity assessment at the DEG-matrix level, eliminating the need for manual, per-gene repetition.

Missing gene expression values are handled before survival stratification according to the selected imputation strategy (Algorithm 1, lines 5–12). When simple imputation is selected,

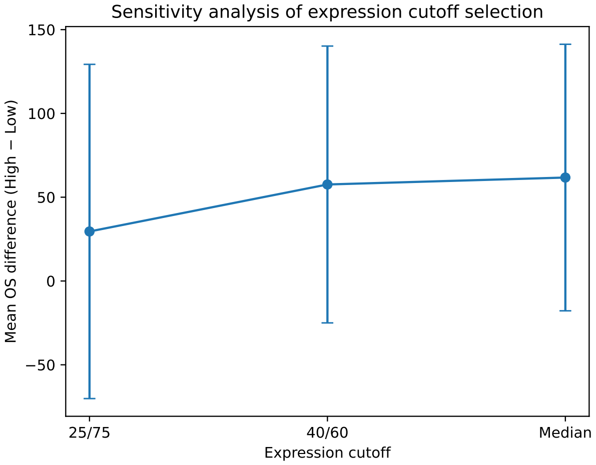

Figure 1 Sensitivity analysis of expression cutoff selection for a representative gene. Differences in mean overall survival (OS) between high- and low-expression groups are shown for median-, 40/60-, and 25/75-percentile-based dichotomizations. Error bars represent pooled standard errors.

DEGAn estimates the chosen gene-wise summary statistic across available samples and uses it to replace missing values, ensuring consistent group definition without discarding samples. Alternatively, KNN-based imputation or complete-case analysis can be applied, depending on the desired balance between computational simplicity, robustness, and data preservation.

A *Bundle* is a custom data structure designed to improve computational efficiency by encapsulating three main components: *(i)* the *rowId* from which it is derived; *(ii)* the expression value of a single DEG observed in the corresponding cell; and *(iii)* symbolic links to the associated annotation data. These links include the overall survival (OS) time, OS status, progression-free survival (PFS) time, PFS status, and the corresponding metastatic status. The use of symbolic links serves a dual purpose: it prevents unnecessary data duplication, thereby reducing memory consumption, and it improves performance by avoiding redundant data movement in main memory. This container-based implementation of the *Bundle* data structure also facilitates extensibility, allowing DEGAn's analytical capabilities to be readily expanded. Algorithm 1, lines 15–22, illustrate the creation and analysis of the *Bundle* data structure.

To further accelerate data analysis, we developed an additional data structure, termed *LifeTimeTable*. The primary purpose of the *LifeTimeTable* is to optimize computation of the product-limit estimator by avoiding repeated reading and processing of the input data, while also providing a thread-safe execution environment during parallel execution. The *LifeTimeTable* acts as a container for specialized objects called *Bundle-Sets*, which offer a compact and efficient representation of individual *Bundles*. Each *Bundle-Set* encodes time-series information by sorting event times, distinguishing between censored and uncensored observations, and computing the group-specific statistics required to evaluate differences in survival experiences. Notably, a single *LifeTimeTable* instance is shared among all worker threads.

To evaluate differences in survival experiences between the detected bundles for each probe, DEGAn applies the *log-rank test* (see Algorithm 1, lines 18–21). The log-rank test assesses the

null hypothesis that the survival curves within a given bundle-set are identical. When more than two expression-defined groups are generated, DEGAn first computes a global log-rank test and then performs pairwise post-hoc comparisons across groups without repetition. For each time interval, the expected number of events of interest (e.g., deaths) is computed and stored for subsequent use. To account for multiple comparisons arising from gene-wise survival testing, DEGAn applies false discovery rate (FDR) correction to all p-values using the Benjamini–Hochberg procedure (see Algorithm 1, line 25).

At the completion of its task, each worker populates the *LifeTimeTable* with the corresponding *Bundle-Sets*, which are stored in ascending order according to their relevance in terms of survival differences. In addition, the *LifeTimeTable* enables workers to retain the values of observed and expected events, as well as counts of patients at risk and events occurring in each time interval, thereby further accelerating survival function estimation. Consequently, the *LifeTimeTable* supports compact representation of DEG-related survival information, reducing memory usage while improving computational efficiency and lowering energy consumption. At the same time, it ensures immediate availability of all data required to compute overall-survival (OS) and progression-free-survival (PFS) curves for each gene. Survival curves are visualized interactively by selecting individual probes. Visualization is straightforward, as it relies on quantities already computed and stored in the *LifeTimeTable* during log-rank test execution. Algorithm 1, line 27, describes this operation.

Complexity analysis

Let G denote the number of genes, S the number of samples, and C the number of available CPU cores. Gene-wise preprocessing requires $O(GS)$ time. For each gene, survival analysis involves a scan over S samples and sorting of event times, yielding a per-gene cost of $O(S \log S)$. Overall, the computational complexity scales as $O(GS \log S)$. Since genes are independent, DEGAn parallelizes computation across available workers, reducing wall-clock time approximately proportionally to the number of computational cores.

Performance evaluation

The performance of DEGAn was evaluated on synthetic gene expression datasets with increasing dimensionality. Two feature sizes were considered, corresponding to 20,000 and 100,000 genes, while the number of samples was progressively increased from 100 to 1,000.

Table 4 Characteristics of the synthetic datasets used to evaluate scalability and resource efficiency.

Num. genes	Num. samples	Feature size	Disk size (MB)
20,000	100	Low-dim	14.2
20,000	250	Low-dim	35.2
20,000	500	Low-dim	70.2
20,000	1,000	Low-dim	140.2
100,000	100	High-dim	71
100,000	250	High-dim	176
100,000	500	High-dim	351
100,000	1,000	High-dim	701

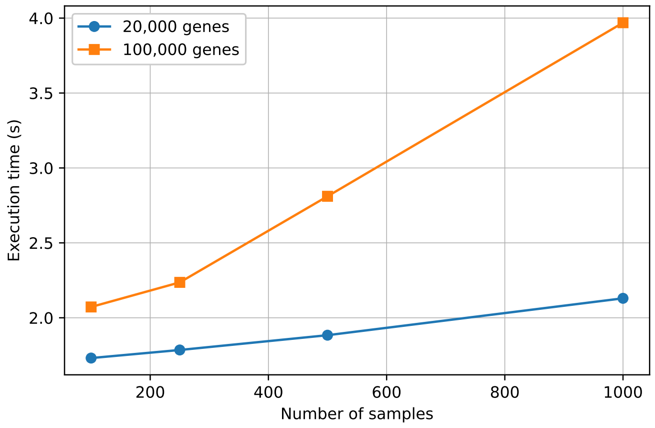

Figure 2 Execution time of DEGAn as a function of the number of samples for datasets with 20,000 and 100,000 genes.

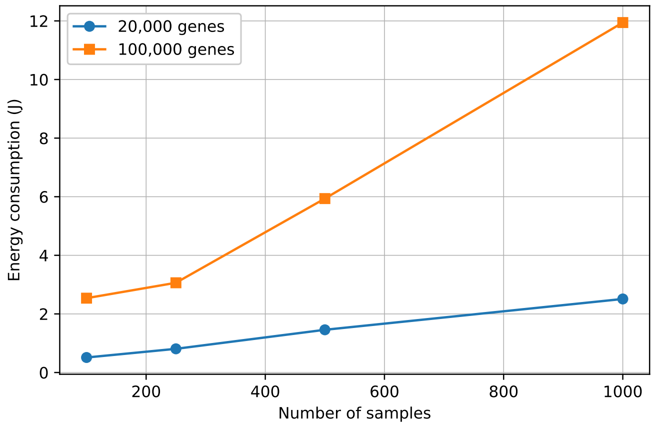

Figure 3 Total energy consumption of DEGAn as a function of the number of samples for datasets with 20,000 and 100,000 genes.

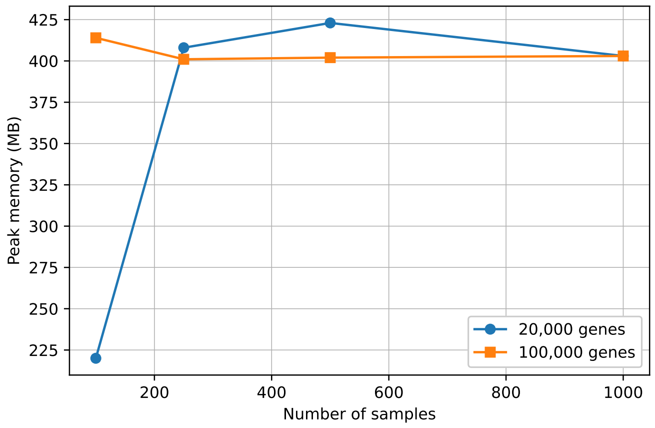

Figure 4 Peak resident set size (RSS) memory usage of DEGAn for datasets with 20,000 and 100,000 genes.

Overall, the performance evaluation shows that DEGAn scales efficiently with the number of samples while remaining robust to increases in feature dimensionality. Execution time and energy

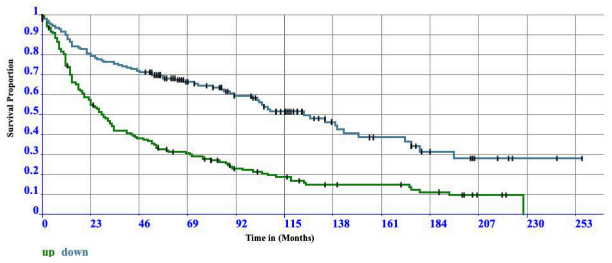

Figure 5 Representative overall survival curve generated by DEGAn for probe set *1552680_a_at*.

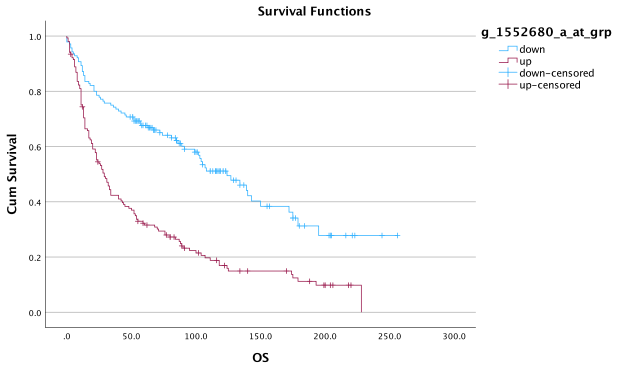

Figure 6 Corresponding overall survival curve generated by SPSS for probe set *1552680_a_at*.

consumption increase moderately as dataset width grows, whereas memory usage remains stable across all tested configurations.

Validation against SPSS

To assess the correctness of DEGAn, results were compared with IBM SPSS Statistics on the GEO dataset GSE30219, which contains 54,680 probes across 307 lung cancer patients together with survival annotations (8). DEGAn processed the dataset directly and produced a ranked list of genes associated with OS. Representative top-ranked probes were exported to SPSS-compatible format and reanalyzed.

The resulting survival curves and log-rank statistics were identical, confirming the correctness of DEGAn's implementation while highlighting its advantages in automation and scalability.

Table 5 Top three probes ranked by DEGAn according to statistical significance derived from log-rank tests comparing overall survival between expression-defined groups.

Probe ID	Gene	p-value
1553015_a_at	RECQL4	1.0037×10^{-3}
228775_at	EMC3	1.1620×10^{-3}
1552680_a_at	KNL1	1.4874×10^{-3}

The biological plausibility of these findings is supported by the literature. RECQL4 has been associated with cancer progression and poor prognosis through mechanisms involving DNA replication and genomic instability (5). EMC3 has been implicated in lung-related pathophysiological processes and tumor-associated pathways (9). KNL1 is a mitotic regulator frequently overexpressed in non-small cell lung cancer and linked to aggressive disease behavior (2).

References

1. Yunshun Chen, Lizhong Chen, Aaron T L Lun, Pedro L Baldoni, and Gordon K Smyth. edger v4: powerful differential analysis of sequencing data with expanded functionality and improved support for small counts and larger datasets. *Nucleic Acids Research*, 53(2):gkaf018, 01 2025.
2. YiRan Dong, Ting Wu, Jiayang Chen, Liang Mo, and Yong You. Knl1 is a lung cancer prognostic biomarker associated with the immune microenvironment. 2024.
3. E. L. Kaplan and Paul Meier. *Journal of the American Statistical Association*, 53(282):457–481, 1958.
4. N Leng and C Kendziorski. Ebseq: An r package for gene and isoform differential expression analysis of rna-seq data. *R package version*, 1(1), 2019.
5. Rongyang Li, Wenhao Yu, Dingxin Wang, Liangyuan Ma, Zhanpeng Tang, Dingqiang Zhu, Zitong Feng, Wenqiang Qi, Hui Tian, and Cun Gao. RECQL4 promotes the malignant progression of lung adenocarcinoma through the YBX1/G3BP1-mediated NF-kB signaling pathway. *Cell Death Discovery*, 12(1):8, January 2026.
6. Michael I. Love, Wolfgang Huber, and Simon Anders. Moderated estimation of fold change and dispersion for RNA-seq data with DESeq2. *Genome Biology*, 15(12):550, December 2014.
7. Pichai Raman, Samuel Zimmerman, Komal S. Rathi, Laurence de Torrenté, Mahdi Sarmady, Chao Wu, Jeremy Leipzig, Deanne M. Taylor, Aydin Tozeren, and Jessica C. Mar. A comparison of survival analysis methods for cancer gene expression rna-sequencing data. *Cancer Genetics*, 235-236:1–12, 2019.
8. Sophie Rousseaux, Alexandra Debernardi, Baptiste Jacquiau, Anne-Laure Vitte, Aurélien Vesin, Hélène Nagy-Mignotte, Denis Moro-Sibilot, Pierre-Yves Brichon, Sylvie Lantuejoul, Pierre Hainaut, Julien Laffaire, Aurélien de Reyniès, David G. Beer, Jean-François Timsit, Christian Brambilla, Elisabeth Brambilla, and Saadi Khochbin. Ectopic activation of germline and placental genes identifies aggressive metastasis-prone lung cancers. *Science Translational Medicine*, 5(186):186ra66–186ra66, 2013.
9. Xiaofang Tang, Wei Wei, Yuqing Sun, Timothy E Weaver, Ernesto S Nakayasu, Geremy Clair, John M Snowball, Cheng-Lun Na, Karen S Apsley, Emily P Martin, et al. Emc3 regulates trafficking and pulmonary toxicity of the sftpc i73t mutation associated with interstitial lung disease. *The Journal of Clinical Investigation*, 134(23), 2024.
